# Triglyceride-glucose index and obstructive sleep apnea: a systematic review and meta-analysis

**DOI:** 10.1186/s12944-024-02005-3

**Published:** 2024-01-08

**Authors:** Amir Hossein Behnoush, Amirmohammad Khalaji, Elina Ghondaghsaz, Mahdi Masrour, Zahra Shokri Varniab, Soheil Khalaji, Alessandro Cannavo

**Affiliations:** 1 School of Medicine, Tehran University of Medical Sciences, Poursina St., Keshavarz Blvd, Tehran, 1417613151 Iran; 2 Non-Communicable Diseases Research Center, Endocrinology and Metabolism Population Sciences Institute, Tehran University of Medical Sciences, Tehran, Iran; 3 Undergraduate Program in Neuroscience, University of British Columbia, Vancouver, BC Canada; 4 Department of Translational Medicine Sciences, Federico II University of Naples, Naples, Italy

**Keywords:** Obstructive sleep apnea, TyG, Triglyceride-glucose index, Systematic review, Meta-analysis

## Abstract

**Background:**

Obstructive sleep apnea (OSA) has a bidirectional association with metabolic syndrome, and insulin resistance (IR). The triglyceride-glucose (TyG) index could be a simply calculated marker of IR in OSA. However, its clinical application appears still limited. Hence, this systematic review and meta-analysis aimed to respond to this question by analyzing all the existing studies showing an association between OSA and the TyG index.

**Methods:**

Four online databases, including PubMed, Scopus, the Web of Science, and Embase were searched for studies evaluating the TyG index in OSA. After screening and data extraction, a random-effect meta-analysis was performed to compare the TyG index in OSA patients vs. healthy controls by calculating standardized mean difference (SMD) and 95% confidence interval (CI) and pooling the area under the curves (AUCs) for diagnosis of OSA based on this index.

**Results:**

Ten studies involving 16,726 individuals were included in the current systematic review. Meta-analysis indicated that there was a significantly higher TyG index in patients with OSA, compared with the healthy controls (SMD 0.856, 95% CI 0.579 to 1.132, *P* < 0.001). Also, TyG had a diagnostic ability for OSA representing a pooled AUC of 0.681 (95% CI 0.627 to 0.735). However, based on the two studies’ findings, no difference between different severities of OSA was observed. Finally, our data showed that the TyG index is a good potential predictor of adverse outcomes in these patients.

**Conclusion:**

Our study revealed that the TyG index is an easy-to-measure marker of IR for assessing OSA, both in diagnosis and prognosis. Our study supports its implementation in routine practice to help clinicians in decision-making and patient stratification.

**Supplementary Information:**

The online version contains supplementary material available at 10.1186/s12944-024-02005-3.

## Introduction

As a common breathing disorder, obstructive sleep apnea (OSA) is characterized by repetitive hypopnea and apnea episodes during sleep time [1]. In 2007, the World Health Organization (WHO) reported more than 100 million subjects as affected by OSA worldwide [2]. However, recent analyses estimated that nearly 1 billion individuals aged 30–69 years worldwide could present this disorder; out of them, almost 425 million patients present with a moderate to severe disease [3]. Hence, OSA represents a rising global health problem with substantial healthcare costs, especially after studies demonstrated that OSA is a risk factor for several non-communicable diseases. Its association with cardiovascular disease (CVD) and cerebrovascular disease has been established and it has been suggested as a major independent predictor of CVD and all-cause mortality [4].

The direct impact of OSA on CVD has been related to the intimate bidirectional association between OSA and metabolic syndrome, insulin resistance (IR), obesity, and type 2 diabetes mellitus (T2DM), all mechanisms involved in CVD development and progression [5–13]. For instance, it has been suggested that OSA induces IR and alters glucose homeostasis which subsequently increases the risk of T2DM. The most likely way it can affect glucose metabolism is via intermittent hypoxia, sleep fragmentation, and sympathetic hyperactivity [13]. On the other hand, metabolic alterations can adversely influence upper airway patency and ventilatory control, which are mechanistic factors for OSA [14, 15]. Moreover, as demonstrated by preclinical studies in animal models, diabetes and IR can lead to an abnormal ventilatory response to hypoxia and hypercapnia reversible by improving insulin sensitivity [13]. In addition, as reported by Llanos et al. [16], IR could confer underlying defects in pharyngeal collapsibility, increasing OSA in obese individuals.

Knowing these facts, quantifying IR in patients with OSA is of very high importance for systemically adverse event risk prediction and therapeutic interventions’ effects monitoring [17]. Even if several methods are often used to assess IR, including the homeostatic model assessment for IR (HOMA-IR), more simple, dimensionless, low-cost tools such as the triglyceride-glucose (TyG) index have been identified and tested [17–19]. The TyG index is proven to be a valuable and reliable IR marker able to assess the CV risk, particularly heart failure, in different populations, from people with diabetes to those with coronary artery diseases [19]. Indeed, a number of studies assessed the association between TyG and OSA, demonstrating that high TyG levels are related to an increased risk of sleep disorders [20]. Despite these premises, there is still a need for determination and consolidation of the possible usage of the TyG index in clinical practice. This study systematically analyzed the possible correlation between the TyG index and OSA, identifying eventual differences in patients with OSA and controls.

## Methods

### Study protocol

This study’s protocol was registered in The International Prospective Register of Systematic Reviews (PROSPERO) on 15 September 2023 (ID: CRD42023460518).

### Eligibility criteria

Studies were included that: (1) measured the TyG index in patients with OSA (and healthy controls): (2) evaluated the discriminatory potential of the TyG index to distinguish OSA patients from non-OSA healthy controls: (3) compared the TyG index between different severities of OSA, and (4) evaluated the ability of TyG index in predicting outcomes in OSA patients. Review articles, case reports, congress abstracts, and non-English abstracts were excluded.

### Search and study selection

The systematic search was performed in four international databases including PubMed, Scopus, the Web of Science, and Embase. The exact search date for all databases was 6 September 2023. The exact queries used in each database are available in Supplementary Table 1. The EndNote software was used to merge records from each database and remove duplicates.

Two reviewers (AK and EG) used titles and abstracts to find relevant studies. Afterward, they used the articles’ full texts to assess the eligibility of studies to be included in this review. Any disagreements were resolved with the opinion of the third reviewer (ZSV). Finally, the lists of references of all included studies were assessed by two researchers (AK and ZSV) for the possibility of missing relevant studies.

### Data extraction and quality assessment

A spreadsheet was designed to extract the required data from each study. Two authors (AK and EG) independently extracted data from included studies and any disagreement was resolved using the original full text. The columns in our spreadsheet were: study name (first author), publication year, conduction year, country of origin, study design (cross-sectional, cohort, etc.), sample size, mean body mass index (BMI), mean age, male percentage, mean apnea-hypopnea index (AHI), TyG levels in different groups, area under the receiver operating characteristic curve (AUC), and main findings.

Quality assessment was performed using the Newcastle Ottawa scale (NOS) [21] in three domains: selection, comparability, and outcome. Based on the NOS system, scores of > 7 were considered “high quality”.

### Statistical analysis

The statistical analyses and visualizations of the analyses were performed in R version 4.2.2 (R Core Team [2021], Vienna, Austria) using the “meta” package [22]. The random effects model with the inverse variance method was used to perform the meta-analysis on the area under the curve (AUC) values. Because of the anticipated heterogeneity among the studies and the marginally different measurement techniques they employed, this model was chosen. This analysis provides a pooled AUC and corresponding confidence interval (CI) for the sum of the studies [23]. The 95% CI was used to calculate the standard error of the AUCs for use in meta-analysis. If CI was not provided, the Hanley and McNeil method was used to calculate the standard error from the AUC value and sample size [24, 25]. The bias-corrected Hedges’ g standardized mean difference (SMD) was used for comparison of the mean TyG index in patients with OSA and controls. We chose Hedges’ g as it accounts for both test and control sample sizes when determining the effect size [26]. To account for expected heterogeneity and measurement differences, the random effect model with the inverse variance method was used to pool the SMD values. Additionally, I2 and tau2 statistics were used to assess heterogeneity in both meta-analyses. To further investigate the sources of heterogeneity among the studies, univariant meta-regression was also performed.

In the case of median and interquartile range reporting, they were converted to the mean and standard deviation using methods developed by Luo et al. [27] and Wan et al. [28]. Furthermore, having an I2 value greater than 50% and a *P* less than 0.05 was defined as a threshold for statistical significance.

## Results

### Characteristics of included studies

**Fig. 1:**
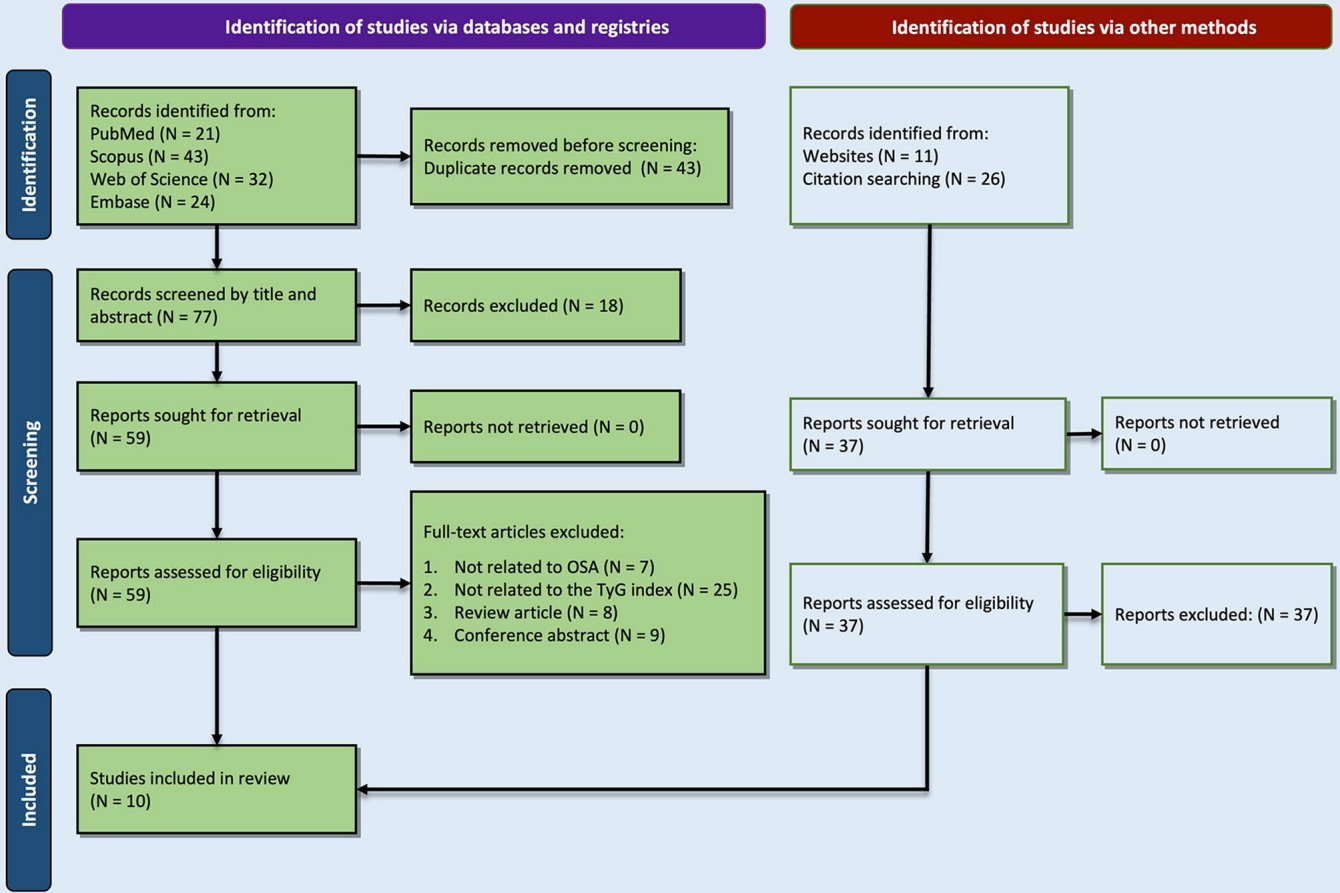
The PRISMA diagram for search and study selection process

An initial screening, using the exact queries shown in Supplementary Table 1, our search yielded 120 records, including 21 from PubMed, 24 from Embase, 32 from Web of Science, and *n* = 43 from Scopus. Of these, 43 studies were removed as duplicates. According to our PRISMA diagram in Fig. 1, another 37 records were identified from citation searches and websites.

All the records (114) went through a deep screening by title/abstract and full-text, and only ten studies were included in the final analysis [18, 20, 29–36]. A total of 16,726 patients with OSA or healthy controls were involved in this review. Most studies (*n* = 6) were conducted in China [20, 30, 31, 34–36], while others were performed in the USA, Hungary, Romania, and Korea [18, 29, 32, 33]. The publication years of the studies were between 2014 and 2023. As shown in Supplementary Table 2, based on the NOS system, studies scored 6 or 7, indicating good quality.

### Mean TyG index: differences between patients with OSA and healthy controls

**Fig. 2:**
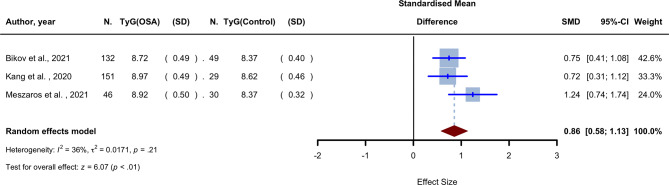
Forest plot showing the random-effect meta-analysis of SMD in comparison of the OSA group vs. control group

Three studies compared the mean TyG index between OSA patients and healthy controls [18, 32, 33]. The studies were conducted in Korea, Hungary, and Romania and were published between 2020 and 2021. The meta-analysis yielded an SMD of 0.856 (95% CI 0.579 to 1.132, *P* < 0.0001, I^2^ = 35.7%) using the random effects model with the inverse variance method (Fig. 2). The result was derived from three mean comparisons involving 329 OSA cases and 108 healthy controls. The weighted mean age of the meta-analysis participants was 49.36, with a male ratio of 63.99%. AHI and BMI mean values were also reported, with weighted averages of 23.76 and 26.51, respectively.

A univariant meta-regression was used to find potential influencing factors and sources of heterogeneity between studies. No statistically significant link between sample size, mean age, male ratio, and effect size was observed in the meta-regression (Table 1). Supplementary Fig. 1 depicts the funnel plot for the SMD meta-analysis. The absence of publication bias is shown by the symmetry of the funnel plot. Furthermore, Egger’s (*P* = 0.345) and Begg’s (*P* = 0.117) tests did not reveal any evidence of publication bias among the included studies.

**Table 1:** Univariant meta-regression results for standardized mean difference (SMD) and area under the curve (AUC) meta-analyses

Moderator	No. of studies	No. of subjects	Meta-regression slope	95% CI	p-value
*Univariant meta-regression for SMD*					
Publication year	3	437	0.239	-0.589 to 1.067	0.571
Sample size	3	437	-0.005	-0.010 to 0.001	0.078
Mean age	3	437	-0.092	-0.659 to 0.474	0.750
Male ratio	3	437	-1.855	-4.756 to 1.046	0.210
AHI	3	437	-0.022	-0.073 to 0.028	0.392
BMI	3	437	0.224	-0.037 to 0.485	0.093
*Univariant meta-regression for AUC*					
Publication year	3	8912	-0.032	-0.049 to -0.015	< 0.001
Sample size	3	8912	-0.000	-0.000 to 0.000	0.290
Mean age	3	8912	0.000	-0.021 to 0.022	0.981
Male ratio	3	8912	-0.029	-0.225 to 0.168	0.775
AHI	2	4883	-0.001	-0.003 to 0.001	0.223
BMI	2	4883	-0.016	-0.043 to 0.010	0.222

SMD: standardized mean difference, CI: confidence interval, AHI: apnea-hypopnea index, BMI: body mass index

### Diagnostic value of the TyG index in OSA patients

**Fig. 3:**
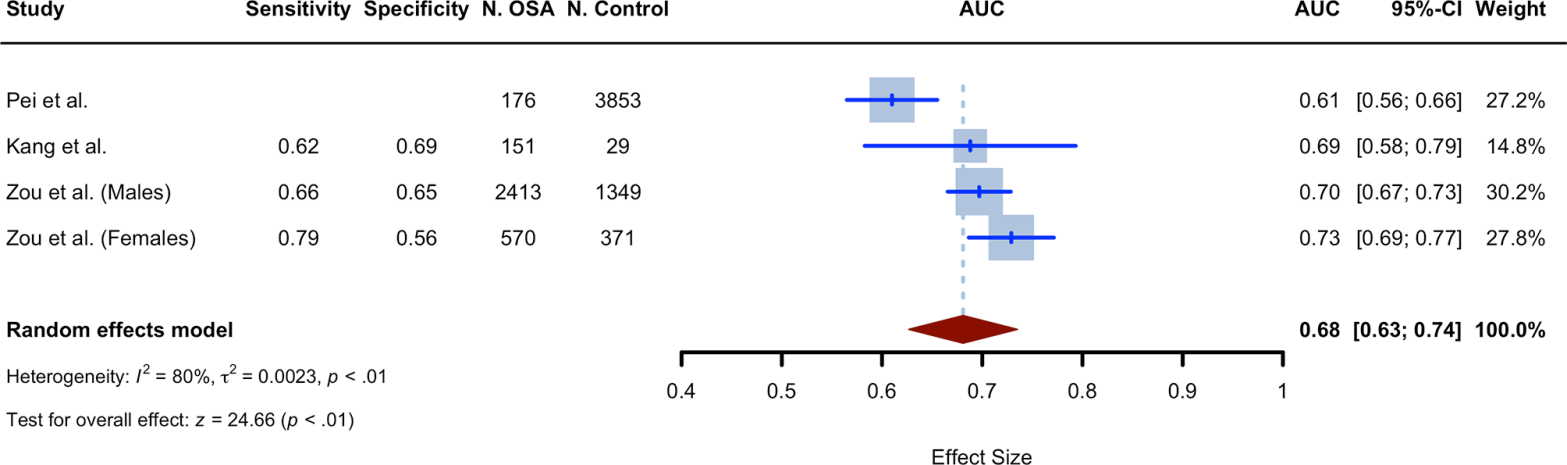
Forest plot showing the random-effect meta-analysis of the TyG index diagnostic AUC values in OSA

Three studies reported diagnostic AUC values [20, 32, 36], with two reporting sensitivity and specificity [32, 36]. Moreover, the study by Zou et al. [36] also reported an AUC value for male and female groups separately. Therefore, four distinct diagnostic AUCs were identified. The studies were published between 2020 and 2023 and were conducted in China and South Korea. Random-effect meta-analysis yielded a pooled AUC value of 0.681 (95% CI 0.627 to 0.735, *P* < 0.0001, I^2^ = 80.4%), calculated using the random effects model with inverse variance method (Fig. 3). This value was derived from four TyG index diagnostic accuracy assessments in OSA, which involved *n* = 3,310 OSA cases and *n* = 5,602 non-OSA controls. The weighted mean age of the included participants in the meta-analysis was 44.27, with a male ratio of 66.94%. AHI and BMI values were also reported in two of the meta-analysis studies. The weighted averages for these parameters were 30.36 and 26.24, respectively.

As shown in Table 1, the univariant meta-regression analysis demonstrated no statistically significant relationship between sample size, mean age, male ratio, and the AUC values. The symmetry of the funnel plot shown in Supplementary Fig. 2 indicates the absence of publication bias. which was also corroborated by Egger’s (*P* = 0.844) and Begg’s tests (*P* = 0.497).

### The association between the TyG index and the risk of OSA

The study by Bianchi et al. [29] evaluated whether there is any association between the levels of TyG index in 302 elderly patients with abdominal aortic aneurysm (AAA). Using the Berlin questionnaire [37], the authors divided the study population into two groups based on their risk of developing OSA: low-risk (*n* = 119) and high-risk (*n* = 183). After adjusting for age, the analyses demonstrated that the high-risk group presented with increased cardiometabolic risk, as shown by several parameters, including insulin, HBA1c, and triglyceride levels, compared to the low-risk group. In addition, a significantly higher TyG index was observed in patients at higher risk for OSA than those at lower risk (8.6 ± 0.5 vs. 8.4 ± 0.5, *P* = 0.002).

### TyG index in different severities of OSA

Two studies compared the TyG index between different severities of the OSA [18, 34]. In this regard, Bikov et al. [18] analyzed the TyG index in 181 volunteers who met the eligibility criteria (Table 2). Of these, 132 were OSA patients who were non-obese and non-diabetic patients and were divided based on the disease severity: 43 mild, 39 moderate, and 50 severe. The remaining 49 out of the 181 volunteers were non-OSA controls. The authors found a significant difference in the TyG index between severe OSA (8.84 ± 0.49) and healthy controls (8.37 ± 0.40) (*P* = 0.03), demonstrating the independent association of this marker with OSA. Moreover, the results supported the ability of the TyG index to predict the disease severity. Indeed, the relationship between the worsening severity of OSA and the rise in the TyG index was significant (adjusted *P* = 0.04). In contrast with these findings, Pan et al. [34] reported that the TyG index was unrelated to OSA disease severity. In particular, the authors analyzed the TyG index in *n* = 94 non-obese men affected by OSA–hypopnea syndrome (OSAHS) divided based on disease severity: *n* = 49 mild to moderate and *n* = 45 severe. Comparing the mean TyG index between these two groups, the authors found no significant difference (mild-to-moderate OSA: 7.27 ± 0.61 vs. severe OSA: 7.31 [IQR 7.00 to 7.77], *P* = 0.305).

**Table 2:** Baseline characteristics and main findings of the included studies evaluating the TyG index in OSA

Study	Year	Location	Population	Sample size	Mean age	Male (%)	AHI (%)	BMI (kg/m^2^)	TyG index	Main Findings
Bianchi et al. [29]	2014	USA	Elderly patients with AAA	302	73.4 (8.6)	86.5	NR	29.6 (6.2)	8.5 (0.5)	Patients with higher risk of OSA had significantly higher TyG index compared to low-risk ones (8.6 ± 0.6 vs. 8.4 ± 0.5, *P* = 0.002)
Bikov et al. [18]	2021	Hungary and Romania	Non-obese, non-diabetic patients with symptoms suggestive for OSA	181	50.3 (15.7)	60.0	19.3 (18.2)	25.9 (3.4)	8.6 (0.5)	The TyG index was significantly higher in patients with OSA compared to controls (8.72 ± 0.49 vs. 8.37 ± 0.40, *P* < 0.01)
Hu et al. [30]	2022	China	Hypertensive patients with OSA and without prior MI	2224	49.5 (10.7)	68.5	21.2 (17.5)	28.4 (3.8)	NR	Higher TyG-WC was associated with a higher risk of first MI in patients with OSA and hypertension (*P* < 0.01)
Jiang et al. [31]	2023	China	Patients with OSA	190	55.3 (13.2)	61.6	41.2 (23.3)	26.1 (3.6)	8.9 (0.6)	TyG index was significantly higher in OSA patients with NAFLD compared to OSA patients without NAFLD (*P* < 0.05)
Kang et al. [32]	2020	Korea	Patients suspected with OSA	180	48.6 (13.8)	73.9	31.6 (28.3)	26.4 (4.1)	8.9 (0.5)	OSA patients had significantly higher levels of TyG index compared to non-OSA individuals (8.97 ± 0.49 vs. 8.62 ± 0.46, *P* < 0.001)
Meszaros et al. [33]	2021	Hungary	Patients suspected with OSA	76	48.9 (14.0)	50.0	15.8 (13.8)	28.2 (8.0)	8.7 (0.4)	TyG index was significantly higher in OSA compared to non-OSA (8.92 ± 0.50 vs. 8.37 ± 0.32, *P* < 0.001)
Pan et al. [34]	2022	China	Non-obese men with OSA	94	46.6 (11.0)	100	31.9 (26.1)	25.2 (2.2)	7.3 (0.6)	No difference was found in TyG levels between severe OSA and mild-to-moderate OSA patients (severe: 7.31 [IQR 7.00–7.77] vs. mild-to-moderate: 7.27 ± 0.61, *P* = 0.305)
Pei et al. [20]	2023	China	Population-based cross-sectional study	4029	46.7	51.4	NR	NR	8.7	No association was found between the TyG index and OSA (adjusted OR 1.559, 95% CI 0.660 to 3.683).
Wei et al. [35]	2021	China	Patients diagnosed with OSA and non-OSA controls	4747	NR	NR	NR	NR	NR	The TyG index was higher in OSA patients compared to non-OSA individuals.
Zou et al. [36]	2020	China	Patients diagnosed with OSA and non-OSA controls	4703	42.3 (13.1)	80.0	31.5 (33.3)	26.4 (3.5)	7.2 (0.6)	Higher TyG levels was associated with higher odds of OSA (men: OR 2.783 [95% CI 1.933 to 4.009], women: OR 3.366 [95% CI 1.975 to 5.737], *P* < 0.001 for both)

TyG: triglyceride-glucose index, OSA: obstructive sleep apnea, AHI: apnea-hypopnea index, AAA: abdominal aortic aneurysms, BMI, body mass index, MI: myocardial infarction, TyG-WC: triglyceride-glucose index-waist circumference, IQR: interquartile range, OR: odds ratio

### TyG index as a predictor marker for adverse outcomes in patients with OSA

Two studies measured the TyG index for the prediction of OSA outcomes [31, 35]. Jiang et al. [31] used TyG to predict nonalcoholic fatty liver disease (NAFLD) in 190 OSA patients. These authors reported that this index was significantly higher in patients with NAFLD than those without (9.13 ± 0.59 vs. 8.69 ± 0.67, *P* < 0.001) and concluded that the TyG index is an independent risk factor for NAFLD in patients with OSA (odds ratio [OR] 1.961, 95% CI 1.03 to 3.73, *P* = 0.04). Finally, the authors reported an AUC of 0.696 [95% CI 0.625 to 0.760] (cutoff = 8.72) for the TyG index in predicting NAFLD in this population. Wei et al. [35] analyzed the correlation between IR and body fat indices, including the TyG index in 3838 patients with OSA (764 normal-weight subjects and 3,074 overweight/obese subjects) and 909 non-OSA controls. The authors reported that the TyG index had a strong association with IR and had a better diagnostic capability in women with OSA than in men. Of note, this study demonstrated AUCs of 0.740 (cutoff 8.54, sensitivity 80.5%, specificity 58.0%, *P* < 0.001) and 0.671 (cutoff 8.99, sensitivity 55.6%, specificity 68.8%, *P* < 0.001) for TyG index in predicting IR in normal-weight and overweight/obese patients with OSA, respectively.

### Modified TyG indices in predicting outcomes in patients with OSA

Two studies used modified TyG indices to predict outcomes in patients with OSA [30, 31]. These modified TyG index versions were calculated by multiplying the TyG index by BMI or waist circumference (WC) and have been proven to be more accurate than the TyG index alone in predicting IR. Based on this premise, Hu et al. [30] used the TyG-WC index to evaluate the association between OSA and myocardial infarction (MI) risk in their observational cohort study. The authors analyzed a population of *n* = 2,224 patients with hypertension and OSA, demonstrating that patients in the highest Quartiles (Q2, 3, and 4) of the baseline TyG-WC index had a greater risk of MI than those in the first quartile. Moreover, they reported that higher TyG-WC (per SD increment) was positively associated with the risk of MI in the OSA population (unadjusted: hazard ratio [HR] 1.54 [95% CI 1.37 to 1.73, *P* < 0.01] and adjusted: HR 1.80 [95% CI 1.49 to 2.18, *P* < 0.01]).

In another study, Jiang et al. [31] used the TyG-WC and TyG-BMI indices to predict NAFLD in patients with OSA. As reported, TyG-BMI and TyG-WC showed AUCs of 0.787 [95% CI 0.722 to 0.843] (cutoff = 0.36) and 0.803 [95% CI 0.739 to 0.857] (cutoff = 0.60), respectively, demonstrating that TyG-WC has the best predictive value for the risk of NAFLD in cases with OSA.

## Discussion

This is the first study systematically assessing the TyG index as a surrogate IR marker in patients with OSA. The precision and comprehensiveness of our search in major databases and independent screening and data extraction processes allowed us to include ten studies and to conclude that (1) patients with OSA had higher levels of TyG index than non-OSA controls; (2) the TyG index showed an AUC of 0.68 in the diagnosis of OSA; (3) higher TyG index can predict adverse outcomes, such as MI and NAFLD in patients with OSA.

OSA is a sleep-related disorder that leads to hypoxia and is currently considered a risk factor for several conditions, including CVD, cerebrovascular disease, dementia, and metabolic syndrome [38–41]. Biomarkers of inflammation [42], endothelial dysfunction [43, 44], renal impairment [45], and cardiac fibrosis showed higher values in OSA patients [46], highlighting the association between OSA and chronic diseases. Moreover, OSA appears to be associated with such disorders in a bidirectional manner. To date, IR has been proven as a pathogenic mechanism connecting OSA to OSA-related diseases, and therefore, many attempts were targeted at finding an appropriate way to measure IR. Notably, among the methods used the TyG index has been proven to be one of the easier-to-dose and cost-effective surrogates of IR with a diagnostic and prognostic value comparable to other IR markers, such as the HOMA-IR or the hyperinsulinemic/euglycemic clamp (HEC) [19, 47, 48]. For instance, in a recent meta-analysis, we demonstrated the potential of TyG in predicting HF incidence in different populations [19]. In line with this notion, Wan and coworkers [49] showed that an augmented TyG is correlated with a higher rate of CVD and stroke incidence. Analogously, Hong et al. [50] by their study supported the utility of the TyG index in predicting dementia risk (including both Alzheimer’s disease and vascular dementia).

As the literature suggests, chronic intermittent hypoxemia is the main pathophysiological way through which OSA could affect IR [51], while sleep fragmentation and deprivation could also play roles in this regard [52]. The key mediator for this association is hypoxia-inducible factor 1 (HIF-1) [53]. However, confounding factors such as the presence of metabolic disorders and obesity should also be taken into consideration [54]. Among the included studies, the study by Bikov et al. [18] adjusted the association between TyG and OSA for BMI, however, even in lean subjects there was a correlation between BMI and TyG index. This highlights the need for adjusting for BMI when researching the association of OSA and metabolic factors. Finally, the role of genetics and epigenetic factors has high importance when interpreting the association of TyG and diseases such as OSA [55].

Our analysis supports the role of the TyG index in diagnosing OSA and the diagnostic ability of this index is comparable to that of other anthropometric indices, such as waist-to-hip ratio, WC, and BMI [56–58], or other lipid indices like the visceral adiposity index (VAI), the lipid accumulation product (LAP), or atherogenic index of plasma (AIP) [59]. Notably, as a result of our analysis, the TyG index is significantly higher in patients with OSA with an SMD of 0.86 (95% CI: 0.58–1.13), confirming the relationship between components of metabolic syndrome (triglyceride and glucose) and OSA, which has also been suggested previously [60, 61].

Our findings have several clinical implications. Other usual methods of IR assessment such as HOMA-IR which is calculated from fasting glucose and insulin levels [62], have several limitations mainly in developing countries in which insulin levels are not routinely measured [63]. Additionally, HOMA-IR has been shown to have limitations in IR evaluation in certain populations [64, 65]. In comparison, the TyG index is highly available and could be calculated from routinely performed laboratory reports [65, 66]. Our findings suggest that patients with OSA could benefit from the assessment of IR, especially through the TyG index, to the extent that it could be added to the routine clinical assessment of patients with OSA even in cases without any other metabolic risk factors.

## Strengths and limitations

The current systematic review and meta-analysis study was the first to investigate the association between the TyG index as a marker of IR and OSA. We showed that there is a significant difference in this index among those with OSA and those without. The comprehensiveness of our search in four databases is another strength of our study. On the other hand, there are four main limitations that should be mentioned in this study. First, including a small number of studies with different populations analyzed might limit our findings and threaten the generalizability of the conclusions. Second, our analyses and reviews were based on observational studies, and therefore, drawing any causal relationship was not possible. Third, the effect of confounding factors such as the use of lipid-lowering medications, special diet, and menopausal status were not considered in individual studies, which could result in an inherent limitation of their pooled results. Finally, despite performing a meta-analysis on AUCs for the diagnostic ability of TyG in OSA, conduction of a meta-analysis for the sensitivity and specificity of this marker was not possible, mainly due to the low number of studies reporting them.

## Conclusion

In the current study, we reviewed and analyzed the role of the TyG index as a diagnostic and prognostic marker in OSA, a condition highly associated with several other disorders, often in a bidirectional manner. Therefore, diagnosis and control of OSA in patients are of extreme importance. As discussed throughout our study, measuring IR could be a valid strategy to assess the risk of OSA and its related disorders. Significantly, along with invasive methods, such as HOMA-IR and HEC, several indirect IR indices have been developed in recent years, such as the ratio of triglycerides to high-density lipoprotein cholesterol (TG/HDLc), and the metabolic score for IR (METS‐IR) [67]. However, based on our analysis, the TyG index or its modified version, such as TyG-WC and TyG-BMI, may represent valuable biomarkers for diagnosing OSA and predicting its related complications risk. Besides, this novel insight adds an important layer to the current understanding of OSA patients’ metabolic dysregulation, indicating the potential clinical use of the TyG index as an IR metric in these patients and those at risk for OSA. Of course, future studies aiming at confirming these findings and comparing the TyG index in different severities of OSA, defined by AHI, would have high value in advancing in this field.

### Electronic supplementary material

Below is the link to the electronic supplementary material.

Supplementary Material 1. Supplementary Tables and Figures

## Data Availability

Not applicable.

## References

[1] Lévy P, Kohler M, McNicholas WT, Barbé F, McEvoy RD, Somers VK (2015). Obstructive sleep apnoea syndrome. Nat Rev Dis Primers.

[2] WHO. Global surveillance, prevention and control of chronic respiratory diseases. 2007.

[3] Benjafield AV, Ayas NT, Eastwood PR, Heinzer R, Ip MSM, Morrell MJ (2019). Estimation of the global prevalence and burden of obstructive sleep apnoea: a literature-based analysis. Lancet Respir Med.

[4] Ge X, Han F, Huang Y, Zhang Y, Yang T, Bai C (2013). Is obstructive sleep apnea associated with cardiovascular and all-cause mortality?. PLoS ONE.

[5] Michalek-Zrabkowska M, Macek P, Martynowicz H, Gac P, Mazur G, Grzeda M et al. Obstructive sleep apnea as a risk factor of insulin resistance in nondiabetic adults. Life (Basel). 2021;11(1). 10.3390/life11010050 PMC7828530 33451031

[6] Yacoub M, Youssef I, Salifu MO, McFarlane SI. Cardiovascular Disease Risk in Obstructive Sleep apnea: an update. J Sleep Disord Ther. 2017;7(1). 10.4172/2167-0277.1000283 PMC5891150 29644149

[7] Kono M, Tatsumi K, Saibara T, Nakamura A, Tanabe N, Takiguchi Y (2007). Obstructive sleep apnea syndrome is associated with some components of metabolic syndrome. Chest.

[8] McArdle N, Hillman D, Beilin L, Watts G (2007). Metabolic risk factors for vascular disease in obstructive sleep apnea: a matched controlled study. Am J Respir Crit Care Med.

[9] Kent BD, McNicholas WT, Ryan S (2015). Insulin resistance, glucose intolerance and diabetes mellitus in obstructive sleep apnoea. J Thorac Dis.

[10] Lam JC, Mak JC, Ip MS (2012). Obesity, obstructive sleep apnoea and metabolic syndrome. Respirology.

[11] Drager LF, Togeiro SM, Polotsky VY, Lorenzi-Filho G (2013). Obstructive sleep apnea: a cardiometabolic risk in obesity and the metabolic syndrome. J Am Coll Cardiol.

[12] Kent BD, Grote L, Ryan S, Pépin JL, Bonsignore MR, Tkacova R (2014). Diabetes mellitus prevalence and control in sleep-disordered breathing: the European Sleep Apnea Cohort (ESADA) study. Chest.

[13] Huang T, Sands SA, Stampfer MJ, Tworoger SS, Hu FB, Redline S (2022). Insulin resistance, hyperglycemia, and risk of developing obstructive sleep apnea in men and women in the United States. Ann Am Thorac Soc.

[14] n den Borst B, Gosker HR, Zeegers MP, Schols AM (2010). Pulmonary function in diabetes: a metaanalysis. Chest.

[15] Lecube A, Simó R, Pallayova M, Punjabi NM, López-Cano C, Turino C (2017). Pulmonary function and sleep breathing: two new targets for type 2 Diabetes Care. Endocr Rev.

[16] Llanos OL, Galiatsatos P, Guzmán-Vélez E, Patil SP, Smith PL, Magnuson T (2016). Pharyngeal collapsibility during sleep is elevated in insulin-resistant females with morbid obesity. Eur Respir J.

[17] Muniyappa R, Lee S, Chen H, Quon MJ (2008). Current approaches for assessing insulin sensitivity and resistance in vivo: advantages, limitations, and appropriate usage. Am J Physiol Endocrinol Metab.

[18] Bikov A, Frent SM, Meszaros M, Kunos L, Mathioudakis AG, Negru AG et al. Triglyceride-glucose index in Non-Diabetic, non-obese patients with obstructive sleep apnoea. J Clin Med. 2021;10(9). 10.3390/jcm10091932 PMC8125770 33947164

[19] Khalaji A, Behnoush AH, Khanmohammadi S, Ghanbari Mardasi K, Sharifkashani S, Sahebkar A (2023). Triglyceride-glucose index and heart failure: a systematic review and meta-analysis. Cardiovasc Diabetol.

[20] Pei H, Li S, Su X, Lu Y, Wang Z, Wu S (2023). Association between triglyceride glucose index and sleep disorders: results from the NHANES 2005–2008. BMC Psychiatry.

[21] Stang A (2010). Critical evaluation of the Newcastle-Ottawa scale for the assessment of the quality of nonrandomized studies in meta-analyses. Eur J Epidemiol.

[22] Balduzzi S, Rücker G, Schwarzer G (2019). How to perform a meta-analysis with R: a practical tutorial. Evid Based Ment Health.

[23] Higgins JP, Thompson SG, Spiegelhalter DJ (2009). A re-evaluation of random-effects meta-analysis. J R Stat Soc Ser a Stat Soc.

[24] Hanley JA, McNeil BJ (1982). The meaning and use of the area under a receiver operating characteristic (ROC) curve. Radiology.

[25] Obuchowski NA, Lieber ML, Wians FH (2004). Jr. ROC curves in clinical chemistry: uses, misuses, and possible solutions. Clin Chem.

[26] Hedges LV (1981). Distribution theory for Glass’s estimator of Effect size and related estimators. J Educational Stat.

[27] Luo D, Wan X, Liu J, Tong T (2018). Optimally estimating the sample mean from the sample size, median, mid-range, and/or mid-quartile range. Stat Methods Med Res.

[28] Wan X, Wang W, Liu J, Tong T (2014). Estimating the sample mean and standard deviation from the sample size, median, range and/or interquartile range. BMC Med Res Methodol.

[29] Bianchi VE, Herbert WG, Myers J, Ribisl PM, Miller LE, Dalman RL (2015). Relationship of obstructive sleep apnea and cardiometabolic risk factors in elderly patients with abdominal aortic aneurysm. Sleep Breath.

[30] Hu J, Cai X, Li N, Zhu Q, Wen W, Hong J (2022). Association between triglyceride glucose Index-Waist circumference and risk of first myocardial infarction in Chinese hypertensive patients with obstructive sleep apnoea: an Observational Cohort Study. Nat Sci Sleep.

[31] Jiang R, Li Y (2023). Value of triglyceride - glucose index combined with obesity index in predicting nonalcoholic fatty liver disease in individuals with obstructive sleep apnea. J Clin Hepatol.

[32] Kang HH, Kim SW, Lee SH (2020). Association between triglyceride glucose index and obstructive sleep apnea risk in Korean adults: a cross-sectional cohort study. Lipids Health Dis.

[33] Meszaros M, Kunos L, Tarnoki AD, Tarnoki DL, Lazar Z, Bikov A. The role of Soluble Low-Density Lipoprotein receptor-related Protein-1 in obstructive sleep apnoea. J Clin Med. 2021;10(7). 10.3390/jcm10071494 PMC8038392 33916750

[34] Pan QY, Li HQ, Gan XY, Chen X, Liu XR, Li JF. Relationship between slow-wave sleep and serum gamma-glutamine transaminase in non-obese men with obstructive sleep apnea-hypopnea syndrome. Sleep Breath. 2022. 10.1007/s11325-022-02775-z 36586074

[35] Wei RB, Gao ZF, Xu HJ, Jiang CP, Li XY, Liu YP (2021). Body Fat Indices as effective predictors of insulin resistance in Obstructive Sleep Apnea: evidence from a cross-sectional and longitudinal study BFI as predictors of IR in OSA. Obes Surg.

[36] Zou J, Wang Y, Xu H, Xia Y, Qian Y, Zou J (2020). The use of visceral adiposity variables in the prediction of obstructive sleep apnea: evidence from a large cross-sectional study. Sleep Breath.

[37] Sharma SK, Vasudev C, Sinha S, Banga A, Pandey RM, Handa KK (2006). Validation of the modified Berlin questionnaire to identify patients at risk for the obstructive sleep apnoea syndrome. Indian J Med Res.

[38] Bikov A, Meszaros M, Kunos L, Negru AG, Frent SM, Mihaicuta S. Atherogenic Index of Plasma in obstructive sleep apnoea. J Clin Med. 2021;10(3). 10.3390/jcm10030417 PMC7865393 33499142

[39] Dong L, Lin M, Wang W, Ma D, Chen Y, Su W (2020). Lipid accumulation product (LAP) was independently associatedwith obstructive sleep apnea in patients with type 2 diabetes mellitus. BMC Endocr Disord.

[40] Lin HJ, Chen PC, Liu YH, Hsu CY. Increasing and high prevalence of moderate to severe obstructive sleep apnea in acute ischemic stroke in Taiwan. J Formos Med Assoc. 2023. 10.1016/j.jfma.2023.09.005 37770283

[41] Ercolano E, Bencivenga L, Palaia ME, Carbone G, Scognamiglio F, Rengo G et al. Intricate relationship between obstructive sleep apnea and dementia in older adults. Geroscience. 2023. 10.1007/s11357-023-00958-4 PMC10828345 37814196

[42] Sanchez-Azofra A, Gu W, Masso-Silva JA, Sanz-Rubio D, Marin-Oto M, Cubero P (2023). Inflammation biomarkers in OSA, chronic obstructive pulmonary disease, and chronic obstructive pulmonary disease/OSA overlap syndrome. J Clin Sleep Med.

[43] Atkeson A, Yeh SY, Malhotra A, Jelic S (2009). Endothelial function in obstructive sleep apnea. Prog Cardiovasc Dis.

[44] Behnoush AH, Khalaji A, Amirkhani N, Pezeshki PS. Diagnostic role of circulating endocan levels in obstructive sleep apnea: a systematic review and Meta-analysis. Angiology. 2023:33197231183087. 10.1177/00033197231183087 37290048

[45] Chuang LP, Lin SW, Lee LA, Chang CH, Huang HY, Hu HC (2019). Elevated Serum Markers of Acute Kidney Injury in patients with obstructive sleep apnea. J Clin Sleep Med.

[46] Khalaji A, Amirkhani N, Sharifkashani S, Behnoush AH (2023). Role of galectin-3 as a biomarker in obstructive sleep apnea: a systematic review and meta-analysis. Sleep Breath.

[47] Luo P, Cao Y, Li P, Li W, Song Z, Fu Z et al. TyG Index performs Better Than HOMA-IR in Chinese type 2 diabetes Mellitus with a BMI < 35 kg/m2: a hyperglycemic clamp validated study. Med (Kaunas). 2022;58(7). 10.3390/medicina58070876 PMC9325243 35888595

[48] Son DH, Lee HS, Lee YJ, Lee JH, Han JH (2022). Comparison of triglyceride-glucose index and HOMA-IR for predicting prevalence and incidence of metabolic syndrome. Nutr Metab Cardiovasc Dis.

[49] Wan Y, Zhang Z, Ling Y, Cui H, Tao Z, Pei J (2023). Association of triglyceride-glucose index with cardiovascular disease among a general population: a prospective cohort study. Diabetol Metab Syndr.

[50] Hong S, Han K, Park CY (2021). The insulin resistance by triglyceride glucose index and risk for dementia: population-based study. Alzheimers Res Ther.

[51] Lindberg E, Theorell-Haglöw J, Svensson M, Gislason T, Berne C, Janson C (2012). Sleep apnea and glucose metabolism: a long-term follow-up in a community-based sample. Chest.

[52] Mesarwi O, Polak J, Jun J, Polotsky VY (2013). Sleep disorders and the development of insulin resistance and obesity. Endocrinol Metab Clin North Am.

[53] Gabryelska A, Szmyd B, Panek M, Szemraj J, Kuna P, Białasiewicz P (2020). Serum hypoxia-inducible factor-1α protein level as a diagnostic marker of obstructive sleep apnea. Pol Arch Intern Med.

[54] Cheng Y, Fang Z, Zhang X, Wen Y, Lu J, He S (2023). Association between triglyceride glucose-body mass index and cardiovascular outcomes in patients undergoing percutaneous coronary intervention: a retrospective study. Cardiovasc Diabetol.

[55] Tramunt B, Smati S, Grandgeorge N, Lenfant F, Arnal JF, Montagner A (2020). Sex differences in metabolic regulation and diabetes susceptibility. Diabetologia.

[56] Wang Y, Mao L, Zhang X (2022). Waist-hip ratio is an independent predictor of moderate-to-severe OSA in nonobese males: a cross-sectional study. BMC Pulm Med.

[57] Lim YH, Choi J, Kim KR, Shin J, Hwang KG, Ryu S (2014). Sex-specific characteristics of anthropometry in patients with obstructive sleep apnea: neck circumference and waist-hip ratio. Ann Otol Rhinol Laryngol.

[58] Kum RO, Kundi FCS, Baklacı D, Kum NY, Güler İ, Yılmaz YF (2018). Predicting severe sleep apnea in patients with complaints: Pulse Oximetry and Body Mass Index. Turk Arch Otorhinolaryngol.

[59] Behnoush AH, Bahiraie P, Shokri Varniab Z, Foroutani L, Khalaji A (2023). Composite lipid indices in patients with obstructive sleep apnea: a systematic review and meta-analysis. Lipids Health Dis.

[60] Drager LF, Lopes HF, Maki-Nunes C, Trombetta IC, Toschi-Dias E, Alves MJ (2010). The impact of obstructive sleep apnea on metabolic and inflammatory markers in consecutive patients with metabolic syndrome. PLoS ONE.

[61] Meszaros M, Tarnoki AD, Tarnoki DL, Kovacs DT, Forgo B, Lee J (2020). Obstructive sleep apnea and hypertriglyceridaemia share common genetic background: results of a twin study. J Sleep Res.

[62] Matthews DR, Hosker JP, Rudenski AS, Naylor BA, Treacher DF, Turner RC (1985). Homeostasis model assessment: insulin resistance and beta-cell function from fasting plasma glucose and insulin concentrations in man. Diabetologia.

[63] Li X, Wang J, Niu L, Tan Z, Ma J, He L (2023). Prevalence estimates of the insulin resistance and associated prevalence of heart failure among United Status adults. BMC Cardiovasc Disord.

[64] Kang ES, Yun YS, Park SW, Kim HJ, Ahn CW, Song YD (2005). Limitation of the validity of the homeostasis model assessment as an index of insulin resistance in Korea. Metabolism.

[65] Vasques AC, Novaes FS, de Oliveira Mda S, Souza JR, Yamanaka A, Pareja JC (2011). TyG index performs better than HOMA in a Brazilian population: a hyperglycemic clamp validated study. Diabetes Res Clin Pract.

[66] Simental-Mendía LE, Rodríguez-Morán M, Guerrero-Romero F (2008). The product of fasting glucose and triglycerides as surrogate for identifying insulin resistance in apparently healthy subjects. Metab Syndr Relat Disord.

[67] Liu XZ, Fan J, Pan SJ (2019). METS-IR, a novel simple insulin resistance indexes, is associated with hypertension in normal-weight Chinese adults. J Clin Hypertens (Greenwich).

