# Supplementary material for: Triglyceride-glucose index and obstructive sleep apnea: a systematic review and meta-analysis
Source: Lipids Health Dis. 2024 Jan 8;23:4. doi: 10.1186/s12944-024-02005-3 (PMC10773018; doi:10.1186/s12944-024-02005-3)
Supplement: Supplementary file 1 — Supplementary Material 1. Supplementary Tables and Figures [file 12944_2024_2005_MOESM1_ESM.docx]

**Supplementary Materials**

***Supplementary Table 1.*** *The search queries and the search results used for each database*

| Query | | Results (No.)  6 September 2023 |
| --- | --- | --- |
| **PubMed** | | |
| #1 | (“lipid ind*”[tiab] OR “TyG”[tiab] OR “triglyceride glucose”[tiab] OR “triglyceride-glucose”[tiab] OR “triglyceride-glucose index”[tiab]) | 3,390 |
| #2 | ("sleep-disordered breathing"[tiab] OR "sleep disordered breathing"[tiab] OR "OSA"[tiab] OR "SAS"[tiab] OR "SDB"[tiab] OR "apnea*"[tiab] OR "apnoea*"[tiab] OR "Apnea"[Mesh] OR "Sleep Apnea, Obstructive"[Mesh]) | 91,058 |
| #3 | #1 AND #2 | 21 |
| **Embase** | | |
| #1 | (“lipid ind*”:ti,ab,kw OR “TyG”:ti,ab,kw OR “triglyceride glucose”:ti,ab,kw OR “triglyceride-glucose”:ti,ab,kw OR “triglyceride-glucose index”:ti,ab,kw) | 4,564 |
| #2 | ('apnea, sleep'/exp OR 'apnea, sleep' OR 'apnoea, sleep'/exp OR 'apnoea, sleep' OR 'nocturnal apnea'/exp OR 'nocturnal apnea' OR 'nocturnal apnoea'/exp OR 'nocturnal apnoea' OR 'obstructive sleep apnea'/exp OR 'obstructive sleep apnea' OR 'obstructive sleep apnea hypopnea syndrome'/exp OR 'obstructive sleep apnea hypopnea syndrome' OR 'obstructive sleep apnea syndrome'/exp OR 'obstructive sleep apnea syndrome' OR 'obstructive sleep apnoea'/exp OR 'obstructive sleep apnoea' OR 'obstructive sleep apnoea hypopnoea syndrome'/exp OR 'obstructive sleep apnoea hypopnoea syndrome' OR 'obstructive sleep apnoea syndrome'/exp OR 'obstructive sleep apnoea syndrome' OR 'obstructive sleep-disordered breathing'/exp OR 'obstructive sleep-disordered breathing' OR 'sleep apnea'/exp OR 'sleep apnea' OR 'sleep apnea syndrome'/exp OR 'sleep apnea syndrome' OR 'sleep apnea syndromes'/exp OR 'sleep apnea syndromes' OR 'sleep apnea, obstructive'/exp OR 'sleep apnea, obstructive' OR 'sleep apnoea'/exp OR 'sleep apnoea' OR 'sleep apnoea syndrome'/exp OR 'sleep apnoea syndrome' OR 'sleep apnoea syndromes'/exp OR 'sleep apnoea syndromes' OR 'sleep apnoea, obstructive'/exp OR 'sleep apnoea, obstructive' OR 'sleep disordered breathing'/exp OR 'sleep disordered breathing') | 109,630 |
| #3 | #1 AND #2 | 24 |
| **Web of Science** | | |
| #1 | (TS=“lipid ind*” OR TS=“TyG” OR TS=“triglyceride glucose” OR TS=“triglyceride-glucose” OR TS=“triglyceride-glucose index”) | 3,728 |
| #2 | (TS=("sleep-disordered breathing") OR TS=("sleep disordered breathing") OR TS=("OSA") OR TS=("SAS") OR TS=("SDB") OR TS=("apnea*") OR TS=("apnoea*")) | 263,799 |
| #3 | #1 AND #2 | 32 |
| **SCOPUS** | | |
| #1 | (TITLE-ABS-KEY(“lipid ind*”) OR TITLE-ABS-KEY(“TyG”) OR TITLE-ABS-KEY(“triglyceride glucose”) OR TITLE-ABS-KEY(“triglyceride-glucose”) OR TITLE-ABS-KEY(“triglyceride-glucose index”)) | 6,013 |
| #2 | (TITLE-ABS-KEY("sleep-disordered breathing") OR TITLE-ABS-KEY("sleep disordered breathing") OR TITLE-ABS-KEY("OSA") OR TITLE-ABS-KEY("SAS") OR TITLE-ABS-KEY("SDB") OR TITLE-ABS-KEY("apnea*") OR TITLE-ABS-KEY("apnoea*")) | 285,125 |
| #3 | #1 AND #2 | 43 |
| ***TOTAL RECORDS*** | | ***120*** |
| ***TOTAL RECORDS AFTER REMOVING DUPLICATES*** | | ***77*** |

***Supplementary Table 2.*** *Qualities of included studies based on NOS*

| **Study** | **Selection** | | | | **Comparability** | **Outcome** | | | **Overall**  **Score** |
| --- | --- | --- | --- | --- | --- | --- | --- | --- | --- |
|  | **Representation of exposed cohort** | **Selection of the non-exposed cohort** | **Ascertainment of exposure** | **Outcome of interest presence** |  | **Assessment of outcome** | **Sufficient length of follow-up** | **Loss to follow-up** |  |
| Bianchi et al. (2014) | * | * | * | * | - | * | - | * | 6 |
| Bikov et al. (2021) | * | * | * | * | - | * | - | * | 6 |
| Hu et al. (2022) | * | * | * | * | - | * | *  (7.15 years) | * | 7 |
| Jiang et al. (2023) | * | * | * | * | - | * | - | * | 6 |
| Kang et al. (2020) | * | * | * | * | - | * | - | * | 6 |
| Meszaros et al. (2021) | * | * | * | * | - | * | - | * | 6 |
| Pan et al. (2022) | * | * | * | * | - | * | - | * | 6 |
| Pei et al. (2023) | * | * | * | * | - | * | - | * | 6 |
| Wei et al. (2021) | * | * | * | * | - | * | *  (6 months) | * | 7 |
| Zou et al. (2020) | * | * | * | * | - | * | - | * | 6 |

**
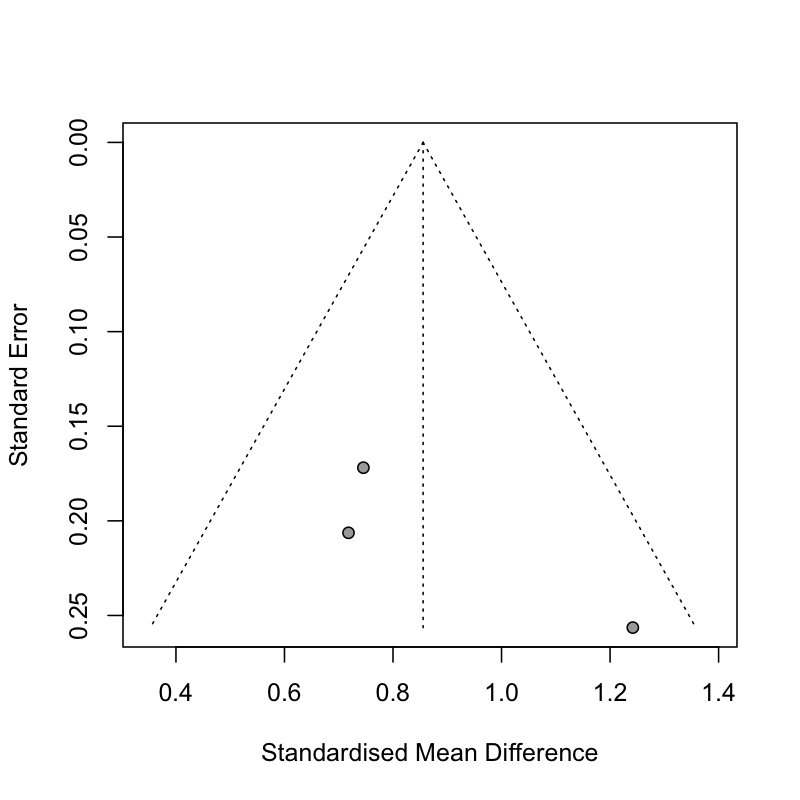
*Supplementary Figure 1.*** *Funnel plot of SMD meta-analysis*

**
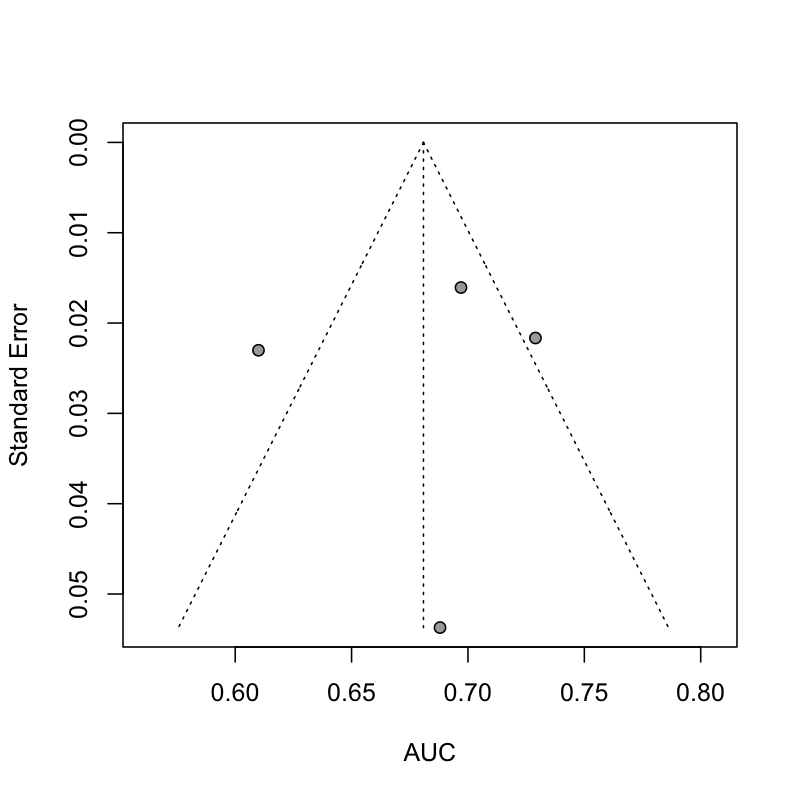
*Supplementary Figure 2.*** *Funnel plot of diagnostic AUCs meta-analysis*
